# *De novo* point mutations in patients diagnosed with ataxic cerebral palsy

**DOI:** 10.1093/brain/awv117

**Published:** 2015-05-16

**Authors:** Ricardo Parolin Schnekenberg, Emma M. Perkins, Jack W. Miller, Wayne I. L. Davies, Maria Cristina D’Adamo, Mauro Pessia, Katherine A. Fawcett, David Sims, Elodie Gillard, Karl Hudspith, Paul Skehel, Jonathan Williams, Mary O’Regan, Sandeep Jayawant, Rosalind Jefferson, Sarah Hughes, Andrea Lustenberger, Jiannis Ragoussis, Mandy Jackson, Stephen J. Tucker, Andrea H. Németh

**Affiliations:** 1 Wellcome Trust Centre for Human Genetics, University of Oxford, OX3 7BN, UK; 2 Universidade Positivo, School of Medicine, Rua Parigot de Souza 5300, 81280-330, Curitiba, Brazil; 3 Centre for Integrative Physiology, Euan MacDonald Centre for Motor Neurone Disease Research, University of Edinburgh, Edinburgh, UK; 4 Nuffield Department of Clinical Neurosciences, University of Oxford, Oxford OX3 9DU, UK; 5 School of Animal Biology, University of Western Australia, Perth, Australia; 6 Section of Physiology & Biochemistry, Department of Experimental Medicine, School of Medicine & Surgery, University of Perugia, P.le Gambuli 1, Edificio D, Piano 106132 San Sisto, Perugia, Italy; 7 Department of Neural and Behavioral Sciences, Pennsylvania State University College of Medicine, Hershey, PA 17033-0850, USA; 8 CGAT Programme, MRC Functional Genomics Unit, Department of Physiology, Anatomy and Genetics, University of Oxford, South Parks Road, Oxford, OX1 3PT, UK; 9 Oxford Medical Genetics Laboratories, Churchill Hospital, Oxford, OX3 7LJ, UK; 10 Fraser of Allander Neurosciences Unit, Royal Hospital for Sick Children, Glasgow G3 8SJ, UK; 11 Department of Paediatrics, Oxford University Hospitals NHS Trust, Oxford, OX3 9DU, UK; 12 Department of Paediatrics, Royal Berkshire Foundation Trust Hospital, Reading, UK; 13 Department of Neuropaediatrics, Development and Rehabilitation, University Children’s Hospital, Inselspital, Bern, Switzerland; 14 Clarendon Laboratory, Department of Physics, University of Oxford, OX1 3PU, UK; 15 OXION Initiative in Ion Channels and Disease, University of Oxford, OX1 3PT, UK; 16 Department of Clinical Genetics, Churchill Hospital, Oxford University Hospitals NHS Trust, Oxford, OX3 7LJ, UK

**Keywords:** cerebral palsy, ataxia, *de novo*, intellectual disability

## Abstract

Cerebral palsy is a sporadic disorder with multiple likely aetiologies, but frequently considered to be caused by birth asphyxia. Genetic investigations are rarely performed in patients with cerebral palsy and there is little proven evidence of genetic causes. As part of a large project investigating children with ataxia, we identified four patients in our cohort with a diagnosis of ataxic cerebral palsy. They were investigated using either targeted next generation sequencing or trio-based exome sequencing and were found to have mutations in three different genes, *KCNC3*, *ITPR1* and *SPTBN2*. All the mutations were *de novo* and associated with increased paternal age*.* The mutations were shown to be pathogenic using a combination of bioinformatics analysis and *in vitro* model systems. This work is the first to report that the ataxic subtype of cerebral palsy can be caused by *de novo* dominant point mutations, which explains the sporadic nature of these cases. We conclude that at least some subtypes of cerebral palsy may be caused by *de novo* genetic mutations and patients with a clinical diagnosis of cerebral palsy should be genetically investigated before causation is ascribed to perinatal asphyxia or other aetiologies.

## Abstract (precis)

Cerebral palsy is commonly attributed to perinatal asphyxia. However, Schnekenberg *et al.* describe here four individuals with ataxic cerebral palsy likely due to *de novo* dominant mutations associated with increased paternal age. Therefore, patients with cerebral palsy should be investigated for genetic causes before the disorder is ascribed to asphyxia.

## Graphical abstract

**Figure:**
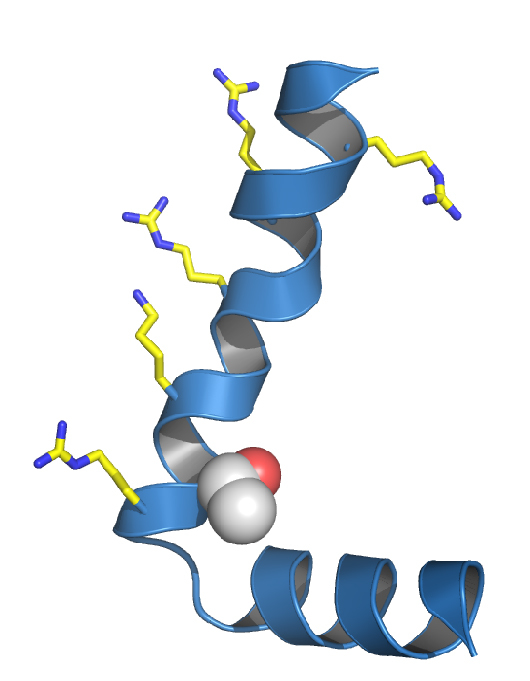
Cerebral palsy is commonly attributed to perinatal asphyxia. However, Schnekenberg *et al.* describe here four individuals with ataxic cerebral palsy likely due to *de novo* dominant mutations associated with increased paternal age. Therefore, patients with cerebral palsy should be investigated for genetic causes before the disorder is ascribed to asphyxia.

## Introduction

The cerebral palsies are defined as a group of permanent disorders of movement and posture that are attributed to non-progressive disturbances that occurred in the developing foetal or infant brain (Rosenbaum *et al.*, 2007). They are classified into clinical subtypes including spastic, dyskinetic, dystonic and ataxic groups, but there may be additional disturbances of cognition, communication and behaviour. Some patients have brain imaging abnormalities, such as focal infarction, brain malformations and periventricular leukomalacia; however, not all children with cerebral palsy have abnormal brain imaging and determining the aetiology in such cases is particularly challenging (O'Shea, 2008; Reid *et al.*, 2013).

Cerebral palsy is a common disorder affecting approximately 1 in 500 of the population in Western industrialized nations (Oskoui *et al.*, 2013), but despite being so common, a proven cause is not always found. Although recent studies have suggested that only 10–20% of cerebral palsy cases can be explained by birth asphyxia (Nelson, 2003; Colver *et al.*, 2014), there remains a widespread belief that obstetric misadventure or even negligence is the main cause of cerebral palsy, and many parents with children diagnosed with cerebral palsy enquire about the possibility of damage during the delivery process. Obstetrics therefore has one of the highest rates of litigation within the medical profession and one of the highest rates of outcome favouring the plaintiff (Jena *et al.*, 2011). Indeed, an internet search for the term ‘cerebral palsy’ provides dozens of legal services for claims of negligence and as a result of this climate of litigation in developed countries the Caesarian section rate (and its complications) has soared in the last 40 years (Nelson, 2003).

Despite major improvements in obstetric and perinatal healthcare, the prevalence of cerebral palsy has remained stubbornly stable (Nelson, 2003), suggesting that intrinsic biological factors rather than extrinsic causes may be an explanation. Genetic factors have been suggested in the context of consanguinity (Moreno-De-Luca *et al.*, 2011, 2012; Kruer *et al.*, 2013), implicating autosomal recessive mutations, but this still fails to explain the frequency of sporadic cases without affected siblings. A recent study of cerebral palsy found a higher rate of rare inherited copy-number variations in cerebral palsy, but the functional significance of these variants was uncertain (McMichael *et al.*, 2013).

In the study presented here, four children were identified who had a working diagnosis of sporadic ataxic cerebral palsy and were found to have *de novo* mutations in three different genes: *KCNC3*, which encodes a voltage-gated potassium channel (K_v_3.3); *ITPR1*, which encodes the receptor for inositol 1,4,5-trisphosphate (IP_3_R); and *SPTBN2*, which encodes β-III spectrin. The likely pathogenicity of the identified mutations was confirmed by using bioinformatics and electrophysiology. This study has significant implications for our understanding of cerebral palsy including its investigation, classification and aetiology.

## Materials and methods

### Genetic studies

Patients were recruited from throughout the UK and Switzerland and consent for participation in the study was obtained according to the Declaration of Helsinki (WMA, 1997) and approved by the Central Oxford Research Ethics Committee and the Research and Development Department of the Oxford Radcliffe Hospitals NHS Trust, Oxford. All patients or their parents provided written consent for the study. Ten cases with a congenital cerebellar ataxia were analysed, the first using targeted capture and next generation sequencing, and the remaining cases were analysed using exome sequencing.

### Targeted sequencing in Case 1

The exonic and 25 base pairs (bp) intronic flanking sequences of 118 genes known to be associated with ataxia in humans or good candidate genes based on function were captured using Agilent SureSelect enrichment and sequenced on the Illumina GAII high-throughput sequencing platform as previously described (Nemeth *et al.*, 2013) (Supplementary material). Sequence reads were aligned to the human reference genome (GRCh37/hg19) and single-nucleotide variants (SNVs) and small indels (insertion and deletions) were identified. Sequence variants were annotated using the Ensembl Variant Effect Predictor (VEP) tool (release 62, April 2011) (Flicek *et al.*, 2011). For each variant, the VEP predicts potential functional consequences (e.g. non-synonymous coding, splice site, intronic effects) and checks if the variant is already present in the single nucleotide polymorphism database (dbSNP, Build 132) (Sherry *et al.*, 2001). All variants were filtered using a previously reported algorithm (Shanks *et al.*, 2013*a*) and analysed using standard pathogenicity prediction programs including PolyPhen-2 (Adzhubei *et al.*, 2010), SIFT (Kumar *et al.*, 2009), MutPred (Li *et al.*, 2009) and the mutation interpretation software Alamut (http://www.interactive-biosoftware.com/). Sanger sequencing was used to confirm variants and parentage by analysing six informative single nucleotide polymorphisms (SNPs) identified as part of the sequencing analysis.

### Next-generation exome sequencing

Instead of targeted capture and sequencing, exome capture was performed in Cases 2–4 plus their parents (‘Trios 2–4’) using the SureSelect Human All Exon kit v5 (Agilent Technologies) and 100 bp paired-end sequencing performed on the Illumina HiSeq 2000 platform. At least 92% of the target region was covered at ×20. Reads were quality trimmed using the FASTX-Toolkit v0.0.13 and then aligned to the 1000 Genomes version of the human reference genome (human_g1k_v37) using the Burrows-Wheeler Aligner (BWA) v0.7.5a. Likely PCR duplicates were removed using Picard v1.106 and BAM files were processed using the Genome Analysis Toolkit (GATK) v2.7.2 software. Single nucleotide variants and indels were jointly called within each trio using the GATK HaplotypeCaller and annotated using SnpEff v3.3 and SnpSift (Cingolani *et al.*, 2012). *De novo* mutations were selected using the Genome Analysis Toolkit Select Variants walker according to previously described criteria (Epi4K Consortium and Epilepsy Phenome/Genome Project, 2013) (Allen *et al.*, 2013). Two additional filters were imposed to select for putative *de novo* variants present at <0.1% frequency in 1000 Genomes individuals and the exome variant server (http://evs.gs.washington.edu/EVS/) and predicted by SnpEff to have a moderate or high impact. Variants were visually inspected using the Integrative Genomics Viewer (IGV) (Thorvaldsdottir *et al.*, 2013) and validated by Sanger sequencing. Nucleotide conservation was estimated using the Genomic Evolutionary Rate Profiling (GERP) (scores: −12.3 = least conserved to 6.17 = most conserved) (Cooper *et al.*, 2005) and PhyloP, which compares the probability of observed substitutions under the hypothesis of neutral evolutionary rate: positive scores suggest constraint (conservation) (Pollard *et al.*, 2010). Effects of amino acid changes were analysed using SIFT (probability of being pathogenic: 0 = highest; 1 = lowest) (Adzhubei *et al.*, 2010) and PolyPhen-2 (probability of being pathogenic: 0 = lowest; 1 = highest) (Sim *et al.*, 2012).

### Confirmation of parentage using exome sequencing data

For Cases 2–4, analysis of parentage was performed using 86 000 exonic Hapmap SNPs, which were genotyped from the exome sequencing data using GATK HaplotypeCaller (McKenna *et al.*, 2010). The non-reference discordance rate (NDR) was calculated by comparing genotype calls for each pair-wise combination of individuals using vcf-compare from the VCFtools suite of software (Danecek *et al.*, 2011). A heatmap depicting NDR values from all combinations was plotted using the ggplot2 package (Wickham, 2009) within the R statistical computing environment (R Core Team (2014). R: A language and environment for statistical computing. R Foundation for Statistical Computing, Vienna, Austria. URL http://www.R-project.org/). Parent-child segregation of SNPs was also investigated for >3000 SNPs where one parent was homozygous non-reference and the other homozygous reference (proband expected to be heterozygous in >99% of cases) and >15 000 SNPs where both parents were homozygous non-reference (proband expected to be homozygous in >99% of cases).

### K_v_3.3 (*KCNC3*) electrophysiological analysis

To examine the functional effects of the T428I mutation in the voltage-gated potassium channel K_v_3.3, the coding region of a sequence-verified human K_v_3.3 (*KCNC3*) cDNA (Source Bioscience) was subcloned into the pBF oocyte expression vector prior to the introduction of the c.1283C>T mutation by SPLICE-based site-directed mutagenesis (Davies *et al.*, 2007). Messenger RNA encoding either wild-type or mutant K_v_3.3 was then transcribed *in vitro* and injected into *Xenopus laevis* oocytes for electrophysiological analysis using a standard two-electrode voltage clamp protocol as previously described (D'Adamo *et al.*, 1998; Imbrici *et al.*, 2000, 2006). Briefly, whole-cell currents for either wild-type or mutant channels were evoked by 200 ms depolarizing commands to +60 mV from a holding potential of –80 mV. For analysis of the heterozygous state, oocytes were co-injected with wild-type and T428I mutant mRNAs (1 ng wild-type and 1 ng T428I) and compared with oocytes injected with either only wild-type mRNA (2 ng) or T428I mutant mRNA (2 ng). Data are presented as the mean ± SEM of 20–50 cells. Statistical significance was determined using an unpaired Student’s *t*-test (**P < 0.001). Relative activation rates were recorded at several voltages and fitted with a single exponential function. The relevant time constants were then calculated and plotted as a function of the test pulse (data are presented as the mean ± SEM of eight cells). The structural homology model of the fourth transmembrane segment (S4 voltage sensor domain), showing the relative position of the T428I mutation was created using the published crystal structure of the related Kv1.2/KCNA2 (potassium voltage-gated channel subfamily A member 2) channel as a template. Within this region, K_v_1.2 exhibits >80% sequence identity with K_v_3.3 (Long *et al.*, 2005).

### β-III spectrin (*SPTBN2*) electrophysiology

Wild-type and R480W mutant rat β-III spectrin cDNA sequences were myc-tagged and cloned into the pRK5 mammalian expression vector. The missense mutation was introduced by site-directed mutagenesis using the pRK5-myc-tagged β-III spectrin as template. Primary rat hippocampal neuronal cultures were prepared from embryonic Day 18 Sprague-Dawley embryos, transfected and current recordings made as previously described (Clarkson *et al.*, 2014). Briefly, cells were co-transfected with constructs expressing wild-type β-III spectrin and GFP or the R480W mutant and DsRed as it was previously shown that the different fluorophores had no effect on sodium currents when co-expressed or expressed alone (Clarkson *et al.*, 2014). Following transfection the cells were mixed and plated together so that recordings could be made from untransfected, wild-type β-III spectrin transfected and R480W mutant transfected cells maintained under identical culture conditions.

## Results

### Clinical summary

**Figure 1:**
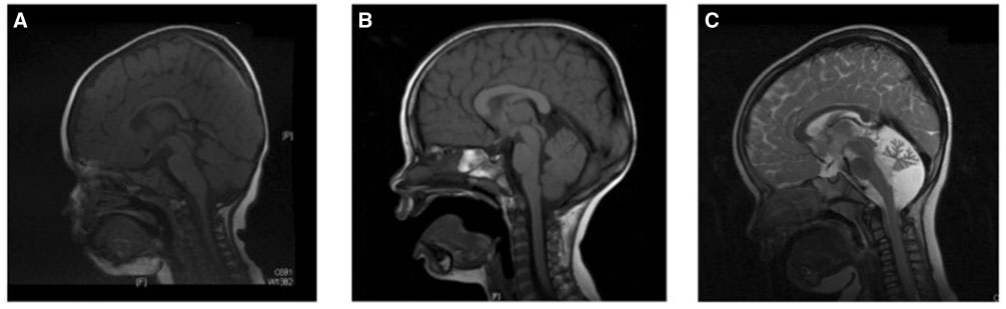
**MRI of the brains of Cases 2, 3 and 4.** (**A** and **B**) Cases 2 and 3 showing a normal brain MRI. (**C**) Case 4 brain MRI shows a small cerebellum, with increased spacing of the cerebellar folia and an enlarged fourth ventricle. This was reported to be cerebellar atrophy, rather than hypoplasia.

**Figure 2:**
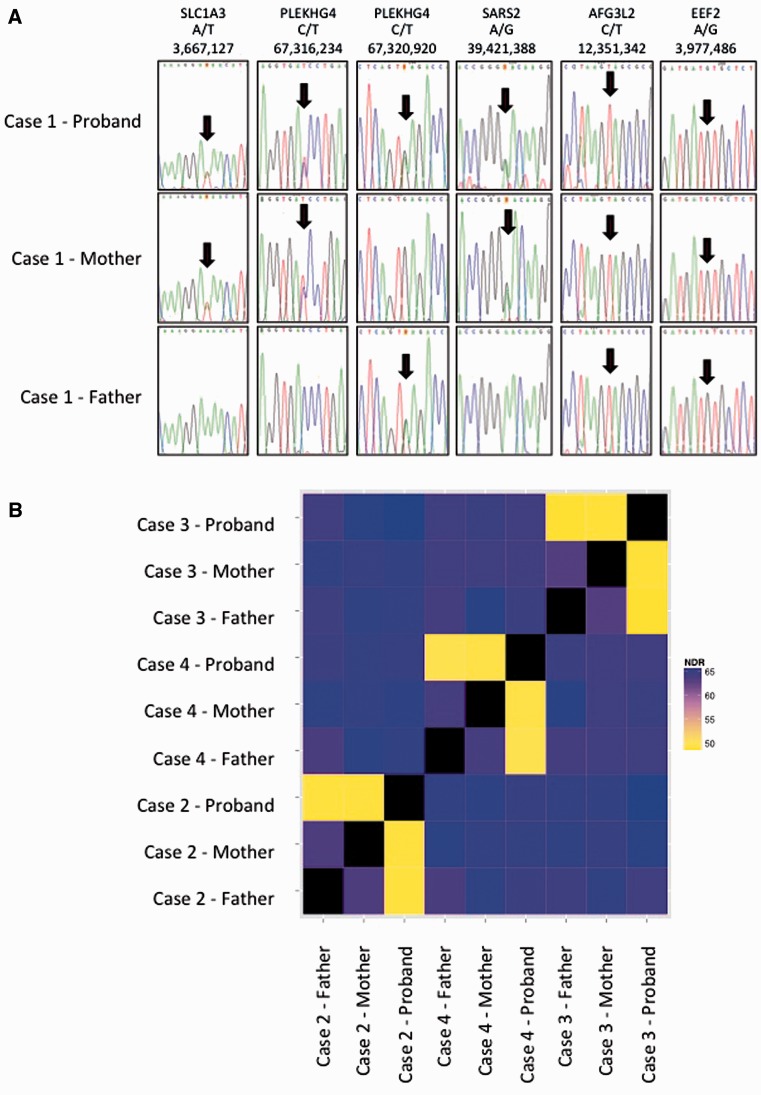
**Confirmation of parentage in Cases 1–4.** (**A**) Sequences of rare SNPs in parents and affected of Case 1 showing consistency with parentage: genes, variant and genomic location (hg19) are shown. (**B**) The non-reference discordance rate (NDR) over 86 000 exonic Hapmap SNPs for Cases 2, 3 and 4. Related individuals show lower discordance (yellow) than unrelated individuals (blue/purple). This analysis confirms that the probands in the study are genetically related to both parents and that parents are not genetically related to each other.

Cases 1–4 were diagnosed with ataxic cerebral palsy based on standard criteria (Smithers-Sheedy *et al.*, 2014), which included the presence of an early ataxic motor disorder with no clinical evidence of a neurodegenerative disorder. All the children were born at term and there were no obvious risk factors for cerebral palsy such as prematurity, low birth weight, infections and hypoglycaemia. No other identifiable causes were found, including neurometabolic disorders. Having identified four cases with *de novo* mutations we reviewed all the ataxia cases in our cohort and identified six additional children who had congenital cerebellar ataxia and who had been analysed using exome sequencing, but who did not have a specific diagnostic label of ataxic cerebral palsy. Case 5 had no brain imaging abnormalities and most closely resembles the other cases in our series. Case 6 had cerebellar vermis hypoplasia and Case 7 had global cerebellar hypoplasia (vermis and cerebellar hemispheres), both these cases were clinically stable. Cases 8 and 9 developed clinical regression by the age of 5 and Case 10 had additional cataracts and an affected sibling. Cases 8–10 would not fulfil the criteria for ataxic cerebral palsy because of the clinical regression and syndromic features and will be presented elsewhere. Table 1 summarizes the clinical details of Cases 1–7 and Fig. 1 shows brain imaging of Cases 2–4. The mutations identified in Cases 1–4 were *de novo*, with parentage confirmed using SNP analysis (Fig. 2 and Supplementary Table 1). Analysis of the raw exome data for Cases 2–4 did not identify any low level somatic mosaicism of the mutant alleles in either parent. No mutations have been found in Cases 5 and 6 and a putative new gene had been found in Case 7, which is currently under investigation and will be presented elsewhere.

**Table 1:** Clinical and genetic data for Cases 1–7

	Case 1	Case 2	Case 3	Case 4	Case 5	Case 6	Case 7
Main clinical features	Ataxia	Ataxia, intellectual disability	Ataxia, intellectual disability	Ataxia, intellectual disability	Ataxia, intellectual disability	Ataxia, intellectual disability, febrile convulsions, low CoQ levels of unknown significance	Ataxia, intellectual disability
Family history	None	None	None	None	None	None	None
Gestation	Term	42 weeks	Term	Term	39 weeks	41 weeks	40 weeks and 3 days
Birth weight (g)	2860	N/A	2730	N/A	3500	3210	4082
Delivery	NVD	NVD	NVD	Induction/EMCS	NVD	NVD	NVD
Previous miscarriages	None	3	None	1	1 TOP (unknown foetal abnormality); 1 ectopic	none	1
Paternal age at conception (years)	40	34	33	36	40	34	33
Evidence of clinical regression	No	No	No	No	No	No	No
Age at most recent review	12	4	10	5	8	6	7
Intellectual disability	Mild	Moderate	Moderate	Moderate	Mild with expressive speech delay (EQ 68)	Mild requiring special school (IQ 81)	Moderate, requiring special school
Brain imaging	Normal	Normal	Normal	Mild cerebellar hypoplasia/ ‘atrophy’	Normal	Mild atrophy of cerebellar vermis	Cerebellar hypoplasia
Mutated gene	*KCNC3*	*ITPR1*	*ITPR1*	*SPTBN2*	None found	None found	Recessive mutations identified in putative novel gene
Nucleotide	1283C>T	1759A>G	4459_4460delinsGA	1438C>T	Not applicable	Not applicable	Under investigation
Protein	T428I	N602D	S1487D	R480W	Not applicable	Not applicable	Under investigation
Nucleotide conservation PhyloP (-14.1-6.4) GERP_RS (-12.3-6.17)	4.64 3.04	3.11 4.98	5.13/6.10 5.71/5.71	3.68 3.55	Not applicable Not applicable	Not applicable Not applicable	Under investigation Under investigation
Amino acid conservation PolyPhen2 (0-1) SIFT (1-0)	0.982 (probably damaging) 0 (Deleterious)	0.958 (probably damaging) 0 (Deleterious)	0.905 (possibly damaging) 0 (Deleterious)	1.0 (Damaging) 0 (Deleterious)	Not applicable Not applicable	Not applicable Not applicable	Under investigation Under investigation
Presence in dbSNP	No	Yes – annotated as pathogenic	No	Yes – annotated as pathogenic	Not applicable	Not applicable	Under investigation
Number of cases in Exome Variant Server	0	0	0	0	Not applicable	Not applicable	Under investigation
Functional domain	Yes – ion selective pore	Yes – IRBIT binding domain	Yes – CA8 binding domain	Yes – Spectrin repeat 2	Not applicable	Not applicable	Under investigation

CoQ = coenzyme Q10; EMCS = emergency Caesarian section; N/A = not available; NVD = normal vaginal delivery; TOP = termination of pregnancy.

### Case 1: T428I in *KCNC3*

Case 1 was reviewed locally in a specialist paediatric neurodisability centre. He is the second child of healthy non-consanguineous parents, with no family history of neurological disorders. He was born at term, weighing 6 lbs 5 oz (2860 g), following an uneventful pregnancy and normal vaginal delivery. He was noted to have early motor delay of sitting, crawling and standing, associated with some delay in cognitive abilities compared with his peers. Investigations included creatine kinase, thyroid function tests, urinary amino and organic acids, chromosomes, genetic testing for Fragile X syndrome and a brain MRI, all of which were normal. After multiple paediatric neurology reviews he was diagnosed as having ataxic cerebral palsy and provided with intensive physiotherapy and occupational therapy support. Further developmental assessments confirmed both the motor abnormalities, with marked disability due to ataxia, in addition to some less prominent cognitive difficulties requiring special support at school. Non-verbal IQ testing gave a score of 90, on the low end of the normal range.

**Figure 3:**
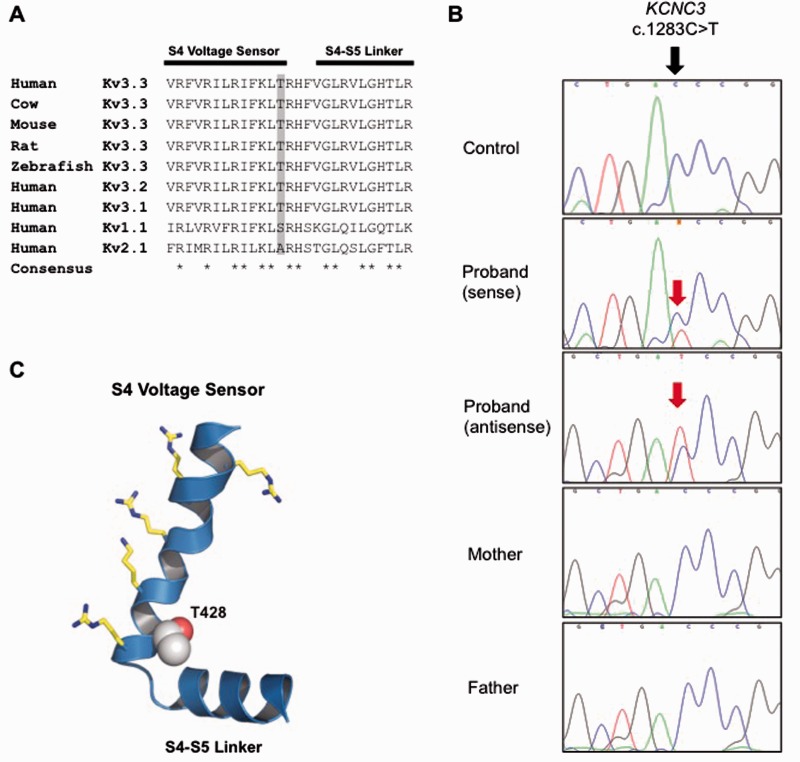
**A novel *de novo* mutation predicted to affect the S4 voltage-sensor of K_v_3.3.** (**A**) The high degree of amino acid conservation (asterisk) in the voltage-sensor S4 helix and S4-S4 linker region of human K_v_3.3 and related species. This region is also highly conserved in the paralogous channels *KCNC1* (K_v_3.1) and *KCNC2* (K_v_3.2). Threonine 428 in *KCNC3* (K_v_3.3) is highlighted in grey and is absolutely conserved between species. (**B**) Sanger sequencing of the patient and parents to show that the heterozygous mutation is *de novo.* (**C**) A structural model of this region in K_v_3.3 with the predicted location of the T428I mutation. The conserved voltage-sensing arginine and lysine residues are also shown.

Three non-synonymous changes were identified using targeted capture and sequencing of 57 known ataxia genes. Of these, only one, a previously unreported *KCNC3* variant c.1283C>T, p.T428I, was predicted to be functionally damaging based on bioinformatic analysis including amino acid conservation (Fig. 3A and Table 1). Bi-directional Sanger sequencing confirmed the next-generation sequencing findings (Fig. 3B) and analyses of other rare SNPs in the proband and his parents were consistent with the stated parentage (Fig. 2), confirming that the mutation arose *de novo* in the patient.

Mutations in *KCNC3* have previously been reported to underlie autosomal dominant spinocerebellar ataxia type 13 (SCA13) (Waters *et al.*, 2006), although some variants of uncertain pathogenic significance have also been reported (Figueroa *et al.*, 2011). Therefore, we further investigated the pathogenicity of the T428I variant by electrophysiological analysis of wild-type and mutant K_v_3.3 potassium channel currents expressed in a heterologous system.

**Figure 4:**
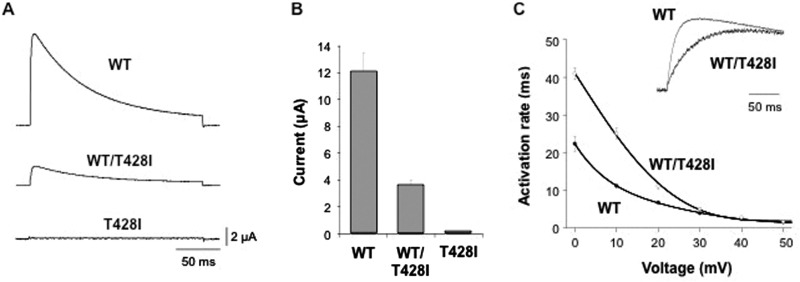
**The T428I mutation affects the functional properties of K_v_3.3.** (**A**) Representative current traces recorded from homomeric wild-type (WT), homomeric mutant (T428I) and heteromeric (WT/T428I) K_v_3.3 channels. The mutant channel exhibits a severe dominant-negative, loss-of-function phenotype. (**B**) Quantifies this dominant negative effect in heteromeric (WT/T428I) channels that mimic the heterozygous state. (**C**) Demonstrates that the residual current in the heterozygous state (WT/T428I) has markedly altered gating properties with much slower rates of activation across a wide range of voltages. The *inset* panel compares representative traces for wild-type and heteromeric (WT/T428I) channels recorded at +60 mV.

The threonine residue at position 428 is a highly conserved amino acid located at the base of the positively charged S4 voltage-sensor helix of the K_v_3.3 potassium channel, a position that has previously been implicated in the control of K_v_ channel activity (Fig. 3C) (Ledwell and Aldrich, 1999; Pathak *et al.*, 2005). Examination of the functional properties of the T428I mutant channel in comparison with wild-type K_v_3.3 clearly demonstrated that the T428I mutant is non-functional (Fig. 4). Moreover, when co-expressed with the wild-type subunit (to mimic the heterozygous state) the T428I mutation exerted a severe dominant negative loss-of-function phenotype, reducing overall channel activity to below 50% of that seen with the wild-type channel alone (Fig. 4B). Importantly, we also observed that the remaining heteromeric channels displayed remarkably slower activation kinetics (Fig. 4C). These results clearly demonstrate that the T428I mutation has a physiologically relevant damaging effect on K_v_3.3 channel function.

### Case 2: N602D in *ITPR1*

**Figure 5:**
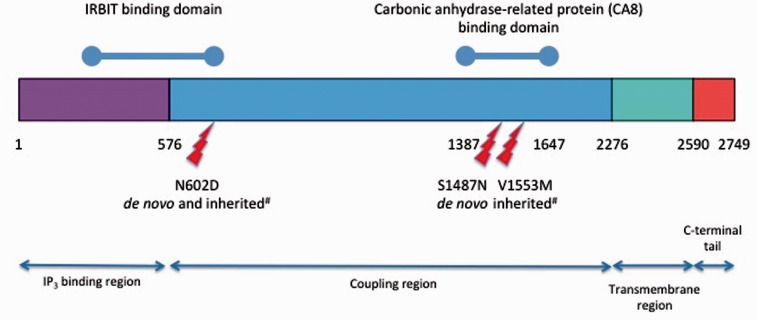
**Location of *ITPR1* missense mutations in functional domains of the IP_3_R protein.** *De novo* N587D and S1487N are described in this paper. Inherited mutations (hash) are reported previously (Huang *et al.*, 2012).

Case 2 is the only child of non-consanguineous parents who was born following difficulties with conception and a single miscarriage. Birth weight was normal and head circumference at birth on the 25th centile. The child was noted to have delayed head control at 6 months, with head nodding and rotatory nystagmus. The abnormal eye movements and head nodding settled and subsequently delay was noted in acquiring developmental milestones. Multiple metabolic investigations were performed but no cause was found. A brain MRI was normal (Fig. 1A). At age 3 she was still not walking independently. Examination revealed hypotonia and ataxia but no long-tract signs. Her head circumference had dropped to the 3–10th centile. She was socially very interactive and could use at least 15 words and will receive special educational support. At almost 4 years old she is making good forward developmental progress; clinical examination reveals ongoing ataxia, she is able to weight-bear, but is not taking steps and has mildly brisk reflexes with tight achilles tendons. In this patient a *de novo* mutation c.1759A>G, p. N602D was detected in *ITPR1* (Fig. 5)*.* Partial gene deletions (usually encompassing the 5’ region) of *ITPR1* are known to cause spinocerebellar ataxia type 15 (SCA15) (van de Leemput *et al.*, 2007) and more recently two missense mutations, N602D and V1553M, were reported in two families with autosomal dominant non-progressive cerebellar ataxia (Huang *et al.*, 2012). Residue N602 is located in the IRBIT (IP_3_R Binding protein released with Inositol 1,4,5-Trisphosphate) binding domain of the *ITPR1* gene product, the IP_3_ receptor (IP_3_R), but little else is known of how this missense mutation causes ataxia. IP_3_R is a receptor for inositol 1,4,5-trisphosphate (IP_3_), which is generated by the hydrolysis of the membrane lipid phosphatidylinositol 4,5-bisphosphate (PIP_2_). IP_3_ binds to the IP_3_R receptor, which is an intracellular ligand-gated calcium ion (Ca^2+^) release channel localized in the endoplasmic reticulum. IP_3_Rs are involved in numerous processes, such as dorsoventral axis formation, synaptic plasticity, neural circuit formation and neuronal dendrite formation (Mikoshiba *et al.*, 1994; Mikoshiba, 2011; Huang *et al.*, 2012). IP_3_R is also modulated by a variety of other proteins including IRBIT, which is a non-enzymatic homologue of *S*-adenosylhomocysteine hydrolase. IRBIT modulates IP_3_R activation by competing with IP_3_ at the IP_3_ binding site, thereby reducing the receptor’s sensitivity to IP_3_ and reducing Ca^2+^ release (Ando *et al.*, 2006). The location of the N602D mutation, its evolutionary conservation (Table 1) and the previous report of an identical mutation in an autosomal dominant cerebellar ataxia family strongly support this mutation being pathogenic.

### Case 3: S1487D in *ITPR1*

Case 3 is the middle child of non-consanguineous parents; the other two children are well. Pregnancy was notable for an increased nuchal thickness, but amniocentesis revealed a normal karyotype. He was born at term by normal vaginal delivery and required no resuscitation. He sat at 12 months, crawled at 13–14 months and walked a few steps before falling at 2 years 5 months. Height, weight and head circumference were all on the 9th–25th centiles. At 4 years he could walk unaided but continued to fall frequently, he had shaky handwriting and could speak in simple sentence structures but had good social interaction. A brain MRI including the cerebellum was normal (Fig. 1B). A complex deletion-insertion in *ITPR1* was identified in this patient at nucleotides c.4459_4460delinsGA, resulting in a missense change p.S1487D. Both nucleotides involved in the mutation are evolutionarily conserved and S1487 is located at a highly conserved position in the CA8 (carbonic anhydrase related protein 8) binding domain of *ITPR1* (Hirota *et al.*, 2003) (Fig. 5 and Table 1) suggesting that this mutation is indeed pathogenic, although at this stage it can only be classified as a ‘possible’ mutation. Similar to IRBIT, CA8 is also a non-enzymatic competitor of IP_3_ that binds to IP_3_R and interestingly, recessive mutations in CA8 cause a congenital ataxia with intellectual disability, possibly acting by disinhibiting the interaction between IP_3_ and the IP_3_R, allowing extra Ca^2+^ to be released from IP_3_-sensitive stores (Turkmen *et al.*, 2009; Kaya *et al.*, 2011)

### Case 4: R480W in *SPTBN2*

Case 4 is the only child of non-consanguineous parents of Mediterranean origin. She was born at term following a normal pregnancy. Delivery was by induction and emergency caesarean section for foetal distress, but she was well following delivery and discharged home within a few days of birth. Within a few weeks she was noted to be head nodding and had unsteady arm movements. An MRI performed abroad was reported to show cerebellar hypoplasia. At 8 months of age she had delayed development although no evidence of regression and was able to sit unaided for short periods. Detailed review at 20 months revealed a normal head circumference, height and weight, although the head circumference was on the 2–9th centile compared to height and weight on the 50th centile, possibly indicating mild relative microcephaly. She had an alternating convergent squint with failure of abduction of the eyes on lateral gaze, deep tendon reflexes were normal and plantars flexor. At 4 years and 10 months of age she was toileting with assistance, had good social interaction with other children, and better receptive than expressive language development. Hearing is normal. Her squint is still present and she continues to be ataxic, being able to stand independently and take a few steps aided. There is no evidence of spasticity or dystonia, she has had no fits, and continues to make forward developmental progress. On this basis a working diagnosis of ataxic cerebral palsy was made. A large set of neurometabolic investigations including renal and liver screen, α-foetoprotein, creatine kinase, transferrin isolelectophoresis, organic acids, very long chain fatty acids, white cell enzymes, quantitative amino acids, and genetic tests including array CGH, mitochondrial mutations, POLG, Friedreichs ataxia, and PLA2G6 were normal. Brain MRI was reported to show cerebellar atrophy (Fig. 1C). Exome sequencing revealed a point mutation c.1438C>T, p.R480W in *SPTBN2* in this patient. The nucleotide is conserved, as is the amino acid at this position (Table 1) and the mutation is predicted as highly likely to be damaging.

**Figure 6:**
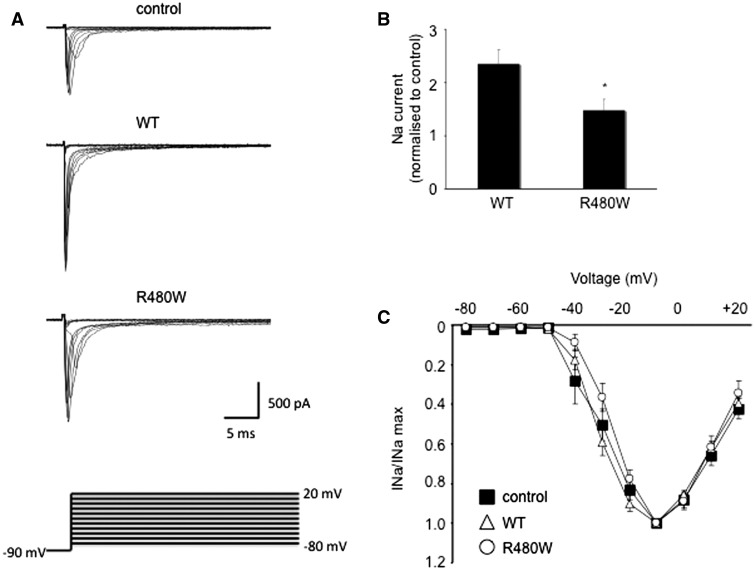
**Peak sodium current enhanced less by R480W than wild-type β-III spectrin.** (**A**) Sodium current traces from representative cells evoked with a series of 50 ms depolorizations from a holding potential of −90 mV to potentials ranging from −80 to +20 mV in 10 mV increments (stimulus protocol shown at bottom). (**B**) Sodium current peak at −10 mV normalized to control cells cultured at same time. (**C**) Current-voltage relationships for control, wild-type (WT) and R480W with current amplitude normalized to peak value. All data are presented as the mean ± SEM (*n* = 5–9 cells from each of three independent cultures; *P* < 0.05).

The same mutation has been reported in another sporadic case of early onset cerebellar ataxia with developmental delay; however in that case no segregation analysis was performed, but the parents were unrelated to each other and healthy, suggesting that the mutation may also have arisen *de novo* (Jacob *et al.*, 2013). This initial case report did not provide any data on the pathogenicity of the mutation, either from segregation or functional analysis. We have previously shown that expression of wild-type βIII spectrin (encoded by *SPTBN2*) can enhance voltage-gated sodium channel currents in cultured hippocampal neurons (Clarkson *et al.*, 2014) in contrast to mutant β-III spectrin. We therefore investigated the effect of this mutation on voltage-gated sodium channel currents in cultured hippocampal neurons and show that sodium currents in the presence of R480W βIII spectrin are lower compared to wild-type βIII spectrin (Fig. 6) providing further evidence for the pathogenicity of this mutation.

## Discussion

This study is the first to report *de novo* mutations in patients with a diagnostic label of cerebral palsy and has important implications for our understanding of this complex condition. Although cerebral palsy is known to be an umbrella diagnostic term with likely multiple causes, the aetiology is often unknown. Our finding of *de novo* mutations provides an explanation for many of the seemingly puzzling features that have been described in the past including the sporadic nature, the highly variable phenotype and the association with increased paternal age (Fletcher and Foley, 1993).

From a clinical perspective, the diagnostic criteria for cerebral palsy were recently and comprehensively reviewed to assist clinicians. These include: a disorder of movement or posture of central origin, a disorder of motor function, no evidence of clinical regression, latest assessment at 4 years of age or older, absence of syndrome/brain anomaly/chromosomal anomaly and no evidence of generalized hypotonia. If the patient fulfils these criteria and, in addition, has ataxia then the diagnosis is ataxic cerebral palsy, although the authors note that differentiating a neurodegenerative cerebellar ataxia can be difficult and recommend investigations to exclude this (Smithers-Sheedy *et al.*, 2014).

Three of our cases clearly fulfilled the clinical criteria for ataxia cerebral palsy as there was no clinical or imaging evidence of regression and there were no syndromic features. However, Case 4 illustrates the complexity of making this diagnosis in young children when based on clinical and radiological features. This patient was born following an emergency Caesarian section for foetal distress and in an era before imaging, the aetiology might have been ascribed to ‘birth injury’. The clinical picture in this case is of a stable cerebellar ataxia, rather than a neurodegenerative disorder and a diagnosis of ataxic cerebral palsy falls within the current definition. Although the imaging shows ‘cerebellar atrophy’ (defined as a cerebellum with initially normal structures in a posterior fossa of normal size, which displays enlarged interfolial spaces in comparison with the foliae secondary to the loss of cerebellar tissue) (Poretti *et al.*, 2008) the lack of clinical regression is not in keeping with a neurodegenerative disorder. Our patient is still young but the previously reported case in the literature reached 12 years of age without evidence of a neurodegenerative disorder, suggesting that this is indeed a clinically stable condition in childhood. Interestingly, there are other cases in the literature with apparent cerebellar atrophy who do not have evidence of clinical regression either and in whom autosomal recessive genetic mutations have been found (Yapici and Eraksoy, 2005; Poretti *et al.*, 2008; Burns *et al.*, 2014). Case 4 certainly warrants specific follow-up for evidence of clinical deterioration and long-term follow-up in patients with these newly defined genetic disorders should greatly assist in determining the prognosis. The difficulty of resolving the apparent contradiction between a clinically stable disorder with cerebellar imaging suggestive of atrophy is also illustrated in the case of *ITPR1* mutations where there do not appear to be consistent genotype–imaging correlations. N602D in *ITPR1* has been reported to cause cerebellar hypoplasia and/or atrophy with long term clinical stability (Huang *et al.*, 2012) but the same *de novo* variant in our Case 2 is associated with normal imaging.

The cases presented here also illustrate the overlap between the diagnostic categories of ataxic cerebral palsy and non-progressive cerebellar ataxia. The latter group of disorders was described in detail prior to the advent of next-generation sequencing (Steinlin, 1998; Steinlin *et al.*, 1998) and include patients with or without cerebellar hypoplasia (as defined in Poretti *et al.*, 2008). Therefore some of these cases would also fulfil the criteria for a diagnosis of ataxic cerebral palsy. The largest group of patients with non-progressive cerebellar ataxia were those of unknown heredity and unknown aetiology (Steinlin, 1998). The clinical features included early hypotonia and motor developmental delay, with occasional spasticity and dystonia. The majority had intellectual disability but the degree of the cognitive impairment could not be predicted by the degree of ataxia or the imaging findings.

A finding of specific interest is that nearly all of our patients also had some degree of intellectual disability. The presence of intellectual disability in cerebral palsy is well known and is estimated to be in the region of 35–50%, therefore the finding of intellectual disability in the majority of our cases of ataxic cerebral palsy is perhaps not surprising. However, there have been no previous reports specifically investigating the association of intellectual disability with ataxic cerebral palsy, and the rate of intellectual disability is higher than might be expected from the overall prevalence rate in cerebral palsy. Although the association between congenital ataxia and intellectual disability has not been emphasized in the literature, recently it has been described in several cases of genetically confirmed congenital ataxia associated with cerebellar atrophy or hypoplasia (Ozcelik *et al.*, 2008; Turkmen *et al.*, 2009; Doi *et al.*, 2011; Bourassa *et al.*, 2012). The neural basis underlying this observation remains uncertain. The concept of the ‘cerebellar cognitive affective syndrome’ has been put forward (Schmahmann and Sherman, 1997) and proposes that the cerebellum has intrinsic cognitive functions. However, in some genetic disorders there is evidence that the genes responsible for cerebellar development are also important in development of the cerebral cortex (and/or hippocampus) (Lise *et al.*, 2012). Further work is required to elucidate the neural basis of this association.

The similarity in clinical phenotypes between our cases suggests that there may be shared molecular mechanisms of pathogenicity. In the case of *KCNC3* and *SPTBN2*, we have shown that the mutations behave as dominant negatives and both of these affect channel function, directly or indirectly. The mechanism of action of missense point mutations in *ITPR1* causing ataxia is unknown, but given the effect of IP_3_R in modulating calcium release it is possible that these also cause dominant negative effects, which further studies will need to elucidate. It is also possible that these three genes affect converging cerebellar neurodevelopmental pathways (Marzban *et al.*, 2014). Genes involved in cerebellar development may also involve cerebral development (Vieira *et al.*, 2010; Martinez *et al.*, 2013) and protein/regulatory networks involved in intellectual disability have been identified in which there is an overlap with proteins affecting cerebellar development (Najmabadi *et al.*, 2011). Whether this hypothesis can be extended to other subtypes of cerebral palsy is unknown, although the extremely wide phenotypic spectrum of cerebral palsy suggests that multiple genes and pathways are likely to be involved.

The clinical phenotype of our patients is unusual for many autosomal dominant spinocerebellar ataxias. Mutations such as R420H in *KCNC3* cause autosomal dominant spinocerebellar ataxia, type 13 (SCA13) with a late onset, progressive disorder. However, two other mutations in *KCNC3*, R423H and P448L do cause a very rare non-progressive early onset autosomal dominant ataxia that is sometimes associated with mild mental retardation and normal imaging, reminiscent of our case and also of the phenotype of ‘cerebral palsy’ (Waters *et al.*, 2006; Figueroa *et al.*, 2010, 2011). It has been suggested that the differences in phenotype are related to the channel properties. The late onset R420H mutation produces a severe dominant negative effect with associated loss of current amplitude, but has little effect on the gating properties of the remaining heteromeric channels. In contrast, the early onset R423H and P448L have dominant negative effects which not only reduce overall current levels, but also alter the properties of channel gating including a hyperpolarized shift in the voltage dependence of activation and slower activation kinetics; this is similar to the effect we observe with the T428I mutation.

Dominant missense or in-frame mutations in *SPTBN2* cause spinocerebellar ataxia type 5 (SCA5) a relatively pure cerebellar disorder with little brainstem or spinocerebellar tract involvement. The disease onset is generally in the third or fourth decade of life, although the age of onset can be as young as 10 years of age and up to 68 years (Bauer *et al.*, 2006). Some juvenile-onset SCA5 patients have bulbar and pyramidal tract dysfunction, in conjunction with a weakened ability to cough, which may shorten lifespan (Ikeda *et al.*, 2006). Cognitive impairment has not been considered a prominent feature in autosomal dominant SCA5. In contrast, autosomal recessive mutations in *SPTBN2* cause a congenital ataxia and cognitive impairment with additional mild spasticity in the two families reported to date (Lise *et al.*, 2012; Elsayed *et al.*, 2014). In both families the mutations are stop codons with presumed loss of function of *SPTBN2* in contrast to the dominant negative effect demonstrated in SCA5 (Clarkson *et al.*, 2010). *SPTBN2* encodes βIII spectrin, which is involved in trafficking and anchoring of crucial neurotransmitter transporters and ion channels to the neuronal cell membranes (Clarkson *et al.*, 2010, 2014; Perkins *et al.*, 2010) and forms a heterotetramer with αII spectrin. The R480W mutation in *SPTBN2* in Case 4 is located in the second spectrin repeat, which is thought to be involved in dimer formation prior to self association into the tetramer and this mutation is therefore likely to have a significant impact on protein function. However, further investigation is required to explain why this specific missense mutation has such a severe phenotype compared with adult-onset SCA5 cases.

Autosomal dominant spinocerebellar ataxia type 15 was first reported to be caused by gene deletions of *ITPR1* (van de Leemput *et al.*, 2007). The mechanism of action has been suggested to be haploinsufficiency, although this has not been proven, and the mechanism of action in missense mutations is unknown. The clinical phenotype in cases involving deletions is of a slowly progressive gait and limb ataxia, often in combination with ataxic dysarthria, titubation, upper limb postural tremor, mild hyperreflexia, gaze-evoked nystagmus, and impaired vestibulo-ocular reflex gain. It is also generally considered to be an adult onset disorder although onset at 7 years has been reported (Storey, 1993). One family has been described with cognitive decline (Castrioto *et al.*, 2011). Missense mutations have only recently been reported (Huang *et al.*, 2012) and the clinical phenotype in the father and daughter reported in that paper is similar to our Case 2: the index case had marked motor and speech delay with sitting at 8 months, only 10 words at 2 years, 4 months and development of complex partial seizures at 5 years of age and the father of the index case was reported to have had developmental delay with independent walking from the age of 5 years and academic difficulties at school (Huang *et al.*, 2012). However, in contrast to our case with normal imaging, the 1-year-old index case was described as having cerebellar hypoplasia with subsequent atrophy by the age of 5. Diffuse cerebellar atrophy was also found in the father of the index case. The father was followed up until aged 45 and there was no evidence of regression in either case. As the mechanism of action is unknown, the explanation for the progressive phenotype in the deletion cases and an early onset but non-progressive disorder in the missense cases remains unknown.

The proportion of ataxic or other forms of cerebral palsy that might be caused by *de novo* mutations is currently unknown, but indirect evidence that this might be a common mechanism includes a higher concordance of cerebral palsy in monozygotic than dizygotic twins, a higher prevalence of congenital anomalies in cerebral palsy patients (which also can be caused by *de novo* mutations), and the previously reported finding of increased paternal age in dyskinetic cerebral palsy (Fletcher and Foley, 1993; Fletcher and Marsden, 1996). A population-based study estimated that 60% of hemiplegic cerebral palsy, 45% of spastic diplegic, and 100% of isolated ataxic cerebral palsy were caused by genetic mutations and interestingly, the mathematical methodology used in the study has been previously validated and successfully applied to the study of individuals with intellectual disabilities, which we now know to be frequently caused by *de novo* mutations (Costeff *et al.*, 1983; Costeff, 2004; Gilissen *et al.*, 2014). A recent study of childhood ataxia has found that compared with the overall prevalence of cerebral palsy (211/100 000), ataxic cerebral palsy is uncommon (10/100 000) but is one of the commonest types of ataxia in childhood (26/100 000). In our modest cohort of 10 patients with congenital ataxia, four had a clinical diagnosis of ataxic cerebral palsy and an additional case (Case 5) was not specifically diagnosed with this, but would fulfil all the clinical and imaging criteria for this. Of these, four patients had *de novo* mutations, and of the four patients who had a normal scan (Cases 1–3, and 5), three had *de novo* mutations.

The frequency with which genetic mutations might be found in cerebral palsy partly depends on the diagnostic terminology that is used. For example, exome sequencing has been reported in cases of ‘cerebral palsy-like encephalopathy’ or ‘masqueraders of cerebral palsy’ and although most examples are autosomal recessive disorders a few *de novo* mutations have been described (Rainier *et al.*, 2006; Lee *et al.*, 2014; Srivastava *et al.*, 2014; Yonekawa *et al.*, 2014). Some of these patients would not fulfil the strict criteria for cerebral palsy but do reflect the challenges clinicians often face in finding an appropriate diagnostic term for young children with highly variable clinical features of unknown aetiology and with an unknown prognosis.

On average each person is thought to harbour ∼75 *de novo* point mutations in their genome, but the majority are in non-coding regions and are unlikely to have deleterious functional consequences. In fact, on average, humans are expected to have only one functionally significant *de novo* point mutation in the exome (Veltman and Brunner, 2012), making the identification of *de novo* mutations the most likely cause of our patient’s motor and intellectual impairments. However, the likelihood that *de novo* mutations are disease-causing can only be determined when such genetic changes are both bioinformatically and functionally tested (Davies *et al.*, 2012; Shanks *et al.*, 2013*b*). Thus, we also provide additional direct experimental evidence for the pathogenicity of several of these mutations by electrophysiological analysis of the mutant proteins. In Cases 1, 2 and 4 the combination of bioinformatics and electrophysiology combined with previous reports provide compelling evidence that these variants are pathogenic. In Case 3, the *de novo* mutation has not previously been reported. The evidence in favour of pathogenicity are that the nucleotide conservation is high, it is predicted as deleterious by SIFT and possibly damaging by PolyPhen-2, in common with the other mutations reported here it is not present in EVS, the variant is located in a functional domain and no other possible variants were identified on exome sequencing. Nevertheless, caution is necessary and at this stage we would classify this as a possible mutation.

Cerebral palsy is a relatively common disorder, but *de novo* mutations are rare genetic events. This paradox can be explained by the reciprocal relationship between the size of the ‘mutational target’ (i.e. the cumulative size of gene loci in which a single large-effect mutation may cause the phenotype) and the frequency of a disease caused by *de novo* mutations (Veltman and Brunner, 2012). In the case of cerebral palsy the ‘mutational target’ is likely to include a huge number of neurodevelopmental genes in which individually rare single *de novo* mutations can lead to an overall high frequency of cerebral palsy within the population. The *de novo* origin of these cerebral palsy cases explains the lack of family history in parents or siblings and, therefore, the lack of a readily apparent genetic cause. This fact may also apply to other cases of cerebral palsy where there are no obvious genetic risks and the risk to siblings of the index case is comparatively low. Advanced paternal age at conception is also associated with *de novo* mutations (de Ligt *et al.*, 2013) and the rate of paternal mutations is estimated to increase by 4.28% per year, which corresponds to doubling every 16.5 years and an increase of two mutations per year (Kong *et al.*, 2012). As much as 20 years ago it was proposed that sporadic cerebral palsy could be associated with such a mechanism (Fletcher and Foley, 1993) and it is therefore interesting to note that the fathers of these four cases all ranged from 34–40 years old at conception.

Our data suggest that cerebral palsy is on a diagnostic and genetic continuum with intellectual disability, disorders of motor development and possibly autistic spectrum disorder, with these latter conditions already known to be caused by *de novo* mutations. Although, hypoxic ischaemic encephalopathy is still likely to explain some cases of cerebral palsy, the identification of genetic causes suggest that this may be less frequent than previously thought. When foetal monitoring was first introduced hypoxic ischaemic encephalopathy was considered to account for up to half of cerebral palsy cases, but this was not supported by subsequent studies and current evidence suggests that only 10–15% of cerebral palsy is caused by birth asphyxia (Nelson, 1988; Graham *et al.*, 2008). There are known risk factors such as low birth weight, prematurity, maternal infections and multiple gestations, but in these cases the cause is relatively obvious. For example maternal fever in labour is associated with an increased risk of cerebral palsy (Grether and Nelson, 1997). However, in many cases, including those presented here, no obvious risk factors were identified and given the implications of this diagnosis to an individual and family, searching for the causation remains a priority. This suggests that great care should be taken in using the term cerebral palsy without extensive investigations to determine aetiology. Indeed in the genomic era, being able to redefine cases according to genetic causation may be extremely useful, allowing for better classifications of cerebral palsy, which in turn should assist management and understanding of prognosis. The identification of *de novo* mutations, which may only have a modest effect on reproductive fitness, also has important implications for genetic counselling of patients, as the risk to the offspring of such individuals will be 50%, whereas the risk to the parents of having further affected children is comparatively low and dependent on the (currently unknown) germline mosaicism rate. Even in cases where there are obvious perinatal risk factors, an underlying genetic mutation still may be present and a thorough search for *de novo* mutations is now clearly warranted before the cause can be ascribed to obstetric misadventure.

The identification of specific, proven pathogenic mutations suggests that a useful way of investigating cerebral palsy patients could be using family based whole-exome or genome sequencing, which efficiently identifies *de novo* mutations in parents and child trio samples (de Ligt *et al.*, 2012, 2013). Exome and whole genome sequencing has revolutionized our ability to investigate patients with rare and complex conditions and its use in clinical practice has been extensively reviewed (Miyatake and Matsumoto, 2014; Schnekenberg and Nemeth, 2014). Although not yet routinely available from all genetic services, there are innovative efforts to test the utility of whole genome sequencing in clinical practice (e.g. http://www.genomicsengland.co.uk/) and whole genome sequencing may yet uncover additional types of *de novo* mutation. Our study has reported point mutations that are comparatively straightforward to detect, but smaller copy number and structural variants (which can be much harder to detect using standard technologies) also may be detectable using whole genome sequencing as recently shown in intellectual disability (Gilissen *et al.*, 2014). Recent large scale studies have particularly demonstrated its efficacy for the identification of *de novo* mutations in developmental disorders that were hitherto intractable using standard genetic investigations (Wright *et al.*, 2015).

In summary, we now provide the first clear evidence that some cases of ataxic cerebral palsy can be caused by *de novo* point mutations and suggest that this finding may be relevant to other subtypes of cerebral palsy as well. The diagnostic overlap between ataxic cerebral palsy, non-progressive cerebellar ataxia and other conditions such as intellectual disability and autistic spectrum disorder raises important questions about the use of the diagnostic term ‘cerebral palsy’, its relationship to perinatal injury and the effect of presumed causation on obstetric practice. Although further work is required to expand the spectrum of mutations causing cerebral palsy, our observations should prompt a major re-evaluation of the aetiology and management of this devastating condition.

## Funding

This work was supported by CNPq (National Council for Scientific and Technological Development), Brazil, to R.P.S.; by the Wellcome Trust (093077) to M.J. and E.P., part of an Australian Research Council (ARC) grants awarded to W.I.L.D. (Future Fellowship, FT110100176; Discovery Project, DP140102117); grants awarded by Telethon Italy, MIUR-PRIN, Ministry of Health (GGP11188, 20108WT59Y_004 and GR-2009-1580433) to M.P.; KF and DS are funded by the Medical Research Council (UK) Computational Genomics Analysis and Training programme (G1000902). Wellcome Trust Grant 075491/Z/04 to J.R.; grants from the Wellcome Trust to S.J.T. (WT084655MA); and awards to A.H.N. from Ataxia UK, Action Medical Research, the Thomas Smith Charity, the Oxford Partnership Comprehensive Biomedical Research Centre funded by the Department of Health National Institute of Health Research (NIHR) Biomedical Research Centre Programme and the Thames Valley Dementias and Neurodegenerative Diseases Research Network (DeNDRoN), UK.

## Supplementary material

Supplementary material is available at *Brain* online.

## Abbreviation

SNP: single nucleotide polymorphism
