# Corrigendum

**DOI:** 10.1093/brain/awv309

**Published:** 2016-01-27

Ricardo Parolin Schnekenberg, Emma M. Perkins, Jack W. Miller, Wayne I. L. Davies, Maria Cristina D’Adamo, Mauro Pessia, Katherine A. Fawcett, David Sims, Elodie Gillard, Karl Hudspith, Paul Skehel, Jonathan Williams, Mary O’Regan, Sandeep Jayawant, Rosalind Jefferson, Sarah Hughes, Andrea Lustenberger, Jiannis Ragoussis, Mandy Jackson, Stephen J. Tucker, Andrea H. Németh. *De novo* point mutations in patients diagnosed with ataxic cerebral palsy. Brain 2015; 138: 1817–32; 10.1093/brain/awv117.

The authors apologize for an incorrect Funding statement, which should read as follows:

This work was supported by CNPq (National Council for Scientific and Technological Development), Brazil, to R.P.S.; by the Wellcome Trust (093077) to M.J. and E.P., part of an Australian Research Council (ARC) grants awarded to W.I.L.D. (Future Fellowship, FT110100176; Discovery Project, DP140102117); grants awarded by Telethon Italy, MIUR-PRIN, Ministry of Health (GGP11188, 20108WT59Y_004 and GR-2009-1580433) to M.P.; K.F. and D.S. are funded by the Medical Research Council (UK) Computational Genomics Analysis and Training programme (G1000902). Wellcome Trust Grant 075491/Z/04 to J.R.; grants from the Wellcome Trust to S.J.T. (WT084655MA); and awards to A.H.N. from Ataxia UK, Action Medical Research, The Henry Smith Charity, the Oxford Partnership Comprehensive Biomedical Research Centre funded by the Department of Health National Institute of Health Research (NIHR) Biomedical Research Centre Programme and the Thames Valley Dementias and Neurodegenerative Diseases Research Network (DeNDRoN), UK.

